# Evolution of physical activity and body weight changes in breast cancer survivors five years after diagnosis – VICAN 2 & 5 French national surveys

**DOI:** 10.1016/j.breast.2021.07.012

**Published:** 2021-07-20

**Authors:** Dominique Rey, Rajae Touzani, Anne-Déborah Bouhnik, Frédérique Rousseau, Adeline Monet, Marie Préau, Marc-Karim Bendiane, Julien Mancini

**Affiliations:** a Aix Marseille Univ, INSERM, IRD, SESSTIM, Economics & Social Sciences Applied to Health & Analysis of Medical Information, CANBIOS Team Labelled League Against Cancer, 232 Bd Sainte Marguerite, 13273, Marseille Cedex, 9, France; b Internal Medicine, Geriatry and Therapeutic Unit, AP-HM Marseille, Hopital Sainte-Marguerite, 19 Avenue Viton, 13009, Marseille, France; c Institut Paoli-Calmettes, SESSTIM U1252, 232 Bd Sainte Marguerite, 13273, Marseille Cedex 9, Marseille, France; d Department of Medical Oncology & Geriatric Coordination Unit for Geriatric Oncology PACA Ouest, Institut Paoli-Calmettes, 232 Bd Sainte Marguerite, 13273, Marseille Cedex 9, Marseille, France; e UR GRePS, Lyon 2 University, 5, Avenue P.Mendès-France, 69676, Bron, France; f APHM, BIOSTIC, Hop Timone, 264 Rue Saint-Pierre, 13005, Marseille, France

**Keywords:** Physical activity, Body weight, Breast cancer, Long-term survivorship

## Abstract

**Background:**

Regular physical activity (PA) and healthy body weight have proven benefits on survival in breast cancer (BC) survivors. We aimed to define predictors of long-term PA and weight gain in a representative sample of BC survivors.

**Methods:**

Data were analysed from 723 women with BC who participated in both the 2012 and 2015 French National VICAN surveys.

**Results:**

Five years after diagnosis, 26.0, 60.6, and 13.4 % of BC survivors reported regular, occasional and no PA, respectively. Moreover, 27.4 % had a weight gain ≥5 kg. In multinomial logistic regressions, regular and occasional PA were both associated with not having depressive disorders, with higher post-traumatic growth, and with a healthy and stable Body Mass Index. Occasional PA was associated with the use of non-conventional medicine, and regular PA with better mental quality of life and normal arm mobility. Weight gain ≥5 kg was associated with younger age, heavier body weight at diagnosis, and lymphedema 5 years after diagnosis.

**Conclusions:**

Mental well-being is associated with successful long-term patient investment in PA. Psychological support and early management of disease sequelae are needed to help ensure BC survivors engage in and maintain healthy lifestyles.

## Highlights

• A quarter of breast cancer survivors at 5 years reported regular physical activity.
• Occasional physical activity was associated with use of non-conventional medicine.
• Mental well-being was associated with long-term investment in physical activity.
• Early sequelae managing may help survivors engage in and maintain healthy lifestyles.

## 1 Introduction

Breast cancer (BC) is the most common cancer in women worldwide, with over 58,000 estimated new cases in France in 2018 [1]. The mortality rate in France has been decreasing for several years thanks to improved cancer screening and therapeutic advances, and the current 5-year survival rate is 88 % [2]. In the growing population of BC survivors, cancer recurrence and poor quality of life (QoL) constitute major public health concerns. Recent meta-analyses showed that physical activity (PA) was generally safe and reduced mortality risk in BC survivors [3,4]. Regular PA has a broad range of benefits, including improvements in cancer-related fatigue, depression, muscle strength and QoL [5,6]. It could also be involved in the pathogenesis and progression of BC [7]. The benefits of PA on survival are even greater when combined with other healthy lifestyle habits [8]. Conversely, excess body weight (BW) at BC diagnosis may worsen prognosis, particularly in post-menopausal women [9]. More specifically, it is associated with a higher risk of experiencing comorbidities, cancer recurrence, second cancer, and mortality [10,11]. Moreover, both weight gain and unexplained weight loss are associated with lower survival rates in women with BC [12].

French and international guidelines encourage cancer survivors to engage in healthy lifestyles and to follow the same recommendations as for primary cancer prevention [13,14]. These include regular PA, healthy BW, a healthy diet, moderating alcohol consumption, and eliminating tobacco use [15]. However, surveys on lifestyle changes and adherence to recommendations following cancer diagnosis have provided conflicting results [16,17]. Using data from the French national VICAN surveys, conducted in 2012 and 2015, we aimed to describe the evolution of PA and BW 5 years after BC diagnosis, and to define predictors of long-term regular and occasional PA, as well as factors associated with significant weight gain in a representative sample of French BC survivors.

## 2 Methods

### 2.1 VICAN surveys

The VICAN surveys were designed to document the life conditions of French cancer survivors two (VICAN 2) and five (VICAN 5) years after diagnosis**.** All French-speaking patients with a primary cancer, aged 18–82 years at diagnosis, diagnosed between January 2010 and December 2011 and registered in one of France's three main Health Insurance schemes (covering over 90 % of the population) were invited to participate. Participants answered a 40-min computer-assisted telephone interview (CATI) in 2011–2012 (VICAN 2) and in 2015–2016 (VICAN 5). The ad hoc questionnaires used covered many topics, from socio-demographic background to cancer sequelae and psychosocial outcomes. Patient data from physicians were also collected and complemented with data from the national health insurance databases (SNIIR-AM) which record information on drugs prescribed and hospital discharge records [18]. A detailed description of the study methodology has been published elsewhere [19]. All participants provided written informed consent before survey enrollment. The methodology was approved by the following three national ethics commissions: the CCTIRS (Advisory Committee on the Processing of Information in the Field of Health Research, registration number 11–143), the ISP (Institute of Public Health, registration number C11-63), and the CNIL (French Commission on Individual Data Protection and Public Liberties, registration number 911290).

### 2.2 Study sample

Our analyses for the present study were based on data from all women diagnosed with BC between January 2010 and December 2011 who participated in both VICAN surveys, and who had no recurrence or cancer progression during the study period. Attrition was compensated by additional inclusions at 5-year, so we only included participants in both VICAN surveys, to be able to study their evolution. Women with a second cancer, those who received chemotherapy, radiotherapy or targeted therapy in 2015–2016, and those admitted to a palliative care unit, were all classified as having progressive cancer and were excluded.

### 2.3 Measurements

#### 2.3.1 Physical activity

VICAN 2 collected information about PA frequency (regular, occasional, none) before BC diagnosis, while VICAN 5 collected information about changes in PA practices since diagnosis (increase, decrease, stopping PA or no change). From these two measurements, we estimated PA frequency five years after BC diagnosis (regular, occasional, none).

#### 2.3.2 BW evolution

We used Body Mass Index (BMI) and BW variations in kilograms as already done in the literature [20,21]. VICAN 2 and VICAN 5 collected, respectively, self-reported BW values before diagnosis and five years post-diagnosis. Using these data, we created a three-category variable for BW evolution in the five years post-diagnosis as follows: significant weight gain (≥5 kg, stable body weight (BW at five years post-diagnosis = BW at diagnosis ±4 kg), and significant weight loss (≥5 kg).

Moreover, body height was recorded in the VICAN 2 questionnaire. Combining it with BW data, we calculated BMI at diagnosis and five years later. BMI values were dichotomized into underweight or normal weight (BMI <24.9 kg/m^2^) and overweight (BMI > 25 kg/m^2^).

#### 2.3.3 Cancer sequelae and psycho-social outcomes

Health-related QoL was measured using the SF12 scale [22]. Cancer-related fatigue was evaluated using three items from the EORTC QLQC30 scale [23], with a score ≥ 40/100 corresponding to clinically significant fatigue [24]. Depression and anxiety were assessed using the Hospital Anxiety and Depression Scale (HADS) [25]. Suspected neuropathic pain was assessed with the 7-item DN4 test [26]. Post-traumatic growth refers to positive psychological change after experiencing traumatic events, including cancer diagnosis [27]. It was assessed with the French validated version of the Post-Traumatic Growth Inventory (PTGI) [28]. There is no threshold value to define positive post-traumatic growth; the higher the score, the more positive the life changes because of cancer. Self-reported BC-specific sequelae (lymphedema, impaired arm mobility, joint pain) and general cancer-related sequelae (from ‘none’ to ‘very severe’) were also documented in the 5-year questionnaire.

#### 2.3.4 Tobacco and alcohol consumption

Self-reported tobacco consumption was collected in both questionnaires. At five years, women were dichotomized into ‘never and former smokers’ vs. ‘current smokers’. Similarly, in the 5-year questionnaire, participants reported alcohol consumption frequency, categorized into ‘never or less than once a month’, ‘one to four times a month’, ‘at least once a week’.

#### 2.3.5 Non-conventional medicine (NCM)

The 5-year questionnaire included an item about use of NCM (“yes” vs “no”).

#### 2.3.6 Medical data

Tumor characteristics and cancer treatment data were collected from each patient's physician(s), while healthcare reimbursement data and hospital discharge data records came from the SNIIR-AM national database. Comorbidity was measured using a score of individual chronic conditions (excluding cancer) based on data from the SNIIR-AM database [29].

#### 2.3.7 Analysis

A weighting procedure was applied to obtain a representative sample of French BC survivors in terms of age, socioeconomic status and cancer progression since diagnosis. Chi-square and ANOVA tests were used to compare survivors according to their PA frequency five years after diagnosis, and to evaluate how their BW changed over the same time period. Multinomial logistic regressions helped identify factors independently associated with PA and BW evolution. In each model, a stepwise procedure was used to select statistically significant factors in the multivariate model (entry threshold p < 0.20). Only factors consistently associated with a p-value ≤0.05 were retained in the latter. All statistical analyses were performed using STATA version 14.0 (StataCorp, College Station, TX, USA).

## 3 Results

Of the 861 women with BC who participated in both VICAN surveys, 136 were excluded from the analyses because of cancer progression between enrolment and the 5-year post-diagnosis evaluation, leaving 723 BC survivors (study sample). Mean age at diagnosis was 50.5 years [range:27–80]. A majority of women (81.4 %) had early stage BC, and only 11.6 % triple negative BC. Most had had surgery (99.4 %), radiotherapy (86.3 %), chemotherapy (55.9 %) and/or adjuvant endocrine therapy (67.0 %). Five years after diagnosis, 21.6 and 45.7 % reported severe and moderate sequelae, respectively, with only 32.6 % receiving related treatment. Fatigue, impaired arm mobility, pain and lymphedema were the most frequently reported late treatment-related symptoms. One third of the study sample had adopted a healthier diet since diagnosis, but 25.5 % reported regular alcohol consumption, 21.1 % were current smokers, and 41.6 % were overweight. Table 1 shows the sample's sociodemographic and clinical characteristics, and self-reported lifestyle habits.

**Table 1:** Characteristics of the study sample (n = 723 breast cancer survivors).

	% - Mean (±SD)
**Age at diagnosis**	50.5 ( ± 11.0)
**Living with a partner**^a^	66.4
**Dependent children**	42.4
**Education level**	
No diploma	6.0
Primary/Secondary	39.8
University	54.2
**Professional situation**^**(e)**^^a^	
Active	58.2
Inactive	11.3
On sick leave or disability	5.1
Retired	25.4
**Perceived financial situation**^**(f**)^^a^	
Comfortable	12.9
Getting by	30.0
Must be careful	40.1
Difficult to make ends meet	17.0
**Suffered from attitudes of rejection or discrimination**^**(g)**^^a^	17.1
**CLINICAL CHARACTERISTICS AND HEALTH STATUS**	
**Tumor stage**	
0	9.7
I/II	71.7
III	7.5
Unknown	11.1
**Breast cancer subtypes**^e^^**(h)**^	
In situ	10.0
ERPR+/Her2-	62.0
Her2+	11.0
Triple negative	11.6
Unclassifiable	5.4
**Surgery**	99.4
**Chemotherapy**	55.9
**Radiotherapy**	86.3
**Breast reconstruction**	28.8
**Adjuvant endocrine therapy**^a^	67.0
**Use of NCM**^a^^b^	31.7
**Comorbidity score at diagnosis**^**(c)**^	0.70 (±0.34)
**Physical Quality of Life**^a^	45.2 ( ± 9.6)
**Mental Quality of life**^a^	43.4 ( ± 11.2)
**Anxiety**^a^^**(b)**^	56.1
**Depressive disorders**^a^^**(b)**^	16.1
**Fatigue (score** ≥ **40)**^a^	55.5
**PTGI score**^a^^**(b)**^	57.4 (±21.6)
**BMI evolution**^**(a)**^^c^	
<27 before diagnosis and <25 five years later	58.4
<25 before diagnosis and ≥25 five years later	11.5
≥25 before diagnosis and 5 years later	30.1
**Body weight evolution**^**(a)**^^a^	
Weight gain ≥5 kg Stable weight	27.4 62.2
Weight loss ≥ 5 kg	10.4
**Self-reported sequelae**^**(d)**^^a^	
Severe	21.6
Moderate	45.7
No sequelae	32.7
**Specific management of sequelae**^d^	32.6
**Lymphedema**^a^ **Impaired arm mobility**^a^	34.6 48.1
**Frequency of pain in the previous fortnight**^**(a)**^^a^	
Often	42.8
Sometimes	39.6
Never	17.6
**Suspected neuropathic pain**^**(a)**^^a^	33.6
**LIFESTYLE HABITS**	
**Physical activity before diagnosis**^**(b)**^	
Yes, regular	36.0
Yes, occasional	52.4
No	11.6
**Change in physical activity**^**(g)**^^a^	
Increase	17.5
Decrease	45.2
Stopped physical activity	5.5
No change	31.8
**Current smoker at diagnosis**	24.5
**Current smoker**^a^	21.1
**Healthier diet**^**(f)**^^a^	34 .1
**Alcohol consumption**^a^	
Never	23.0
Once to 4 times a month	51.5
At least once a week	25.5

a Situation 5 years after breast cancer diagnosis.

b NCM: non-conventional medicine.

c BMI: body mass index; <25 = underweight or normal weight; ≥25 = overweight.

d Among women who reported severe or moderate sequelae 5 years after diagnosis.

e ERPR+: estrogen receptor positive and/or progesterone receptor positive; Her2: Human epidermal growth factor receptor 2 Number of missing values: 2 (a), 3 (b), 4 (c), 6(d),7(e), 9 (f), 10 (g), 64 (h).

### 3.1 Physical activity five years after diagnosis

Five years after cancer diagnosis, 26.0, 60.6 and 13.4 % of 710 women (missing data n = 13) reported regular, occasional and no PA, respectively. Near half of these (45.2 %) reported less frequent PA than before diagnosis, while 5.5 % had stopped PA completely (Table 1). Pre-diagnosis PA was associated with PA at five years (Table 2). Table 2 details other factors associated with PA at five years in the univariate analysis. PA frequency increased with education level. A similar gradient was found for PA frequency and financial situation, professional activity, adopting a healthier diet, and maintaining a healthy BMI. PA was significantly associated with fewer depressive disorders, fewer physical limitations, fewer self-reported sequelae, a lower comorbidity score, higher levels of physical and mental QoL, and higher scores of PTGI. In the multinomial logistic regression model, regular PA five years after diagnosis was independently associated with better mental QoL, no depressive disorders, a healthy and stable BMI since diagnosis, a higher PTGI score, and normal arm mobility. Occasional PA was associated with no depressive disorders, a healthy and stable BMI since diagnosis, a higher PTGI score, and the use of NCM (Table 3).

**Table 2:** Factors associated with physical activity five years after breast cancer diagnosis – VICAN 5 survey (n = 710 in univariate analysis).

		Regular PA (%) n = 185	Occasional PA (%) n = 430	No PA (%) n = 95	p-value
Age at diagnosis	*Mean (SD)*	49.7 (9.9)	50.5 (11.1)	50.7 (11.4)	0.647
Education level	No diploma	3.6	5.5	13.3	**0.015**
	Primary/Secondary	35.9	39.6	48.0	
	University	60.5	54.9	38.7	
Professional situation	Active	67.8	59.4	36.9	< **0.001**
	Inactive, on sick leave or disability	7.0	16.8	34.1	
	Retired	25.2	23.8	29.0	
Perceived financial situation	Comfortable/getting by	50.9	41.8	33.1	**0.041**
	Must be careful/Difficult to make ends meet	49.1	58.2	66.9	
Suffered from attitudes of rejection or discrimination		12.1	19.1	19.2	0.197
Breast cancer subtypes∗∗	In situ	8.9	10.2	11.3	0.850
	ERPR+/Her2-	63.9	62.0	62.3	
	Her2+	12.0	9.8	15.3	
	Triple negative	10.4	12.5	5.9	
	Unclassifiable	4.8	5.5	5.2	
Cancer treatment	Chemotherapy	57.1	55.7	56.4	0.962
	Radiotherapy	84.3	87.4	85.3	0.629
	Adjuvant endocrine therapy	67.4	68.2	67.2	0.979
Breast reconstruction		29.8	31.7	17.3	0.058
Comorbidity score	*Mean (SD)*	0.67 (0.38)	0.68 (0.32)	0.80 (0.34)	**0.03**
Use of NCM		33.7	33.8	18.6	**0.04**
Physical Quality of Life	*Mean (SD)*	49.2 (8.7)	44.7 (9.1)	41.0 (9.9)	< **0.001**
Mental Quality of life	*Mean (SD)*	47.0 (10.3)	42.9 (10.9)	38.7 (11.7)	< **0.001**
Anxiety		50.5	56.9	64.1	0.169
Depressive disorders		6.0	15.1	42.2	< **0.001**
PTGI score (0–105)		61.8 (18.9)	58.0 (21.5)	47.3 (24.0)	< **0.001**
Fatigue (score ≥ 40)		40.7	59.6	66.4	< **0.001**
Lymphedema		23.9	38.2	44.2	**0.004**
Impaired arm mobility		30.7	51.7	75.5	< **0.001**
Pain in the previous 15 days	Often	31.7	44.6	53.8	**0.002**
	Sometimes	42.0	38.8	41.1	
	Never	26.3	16.6	5.1	
Suspected neuropathic pain (DN4)		24.1	36.2	42.5	**0.002**
Joint pain in the previous 7 days		73.6	81.5	84.2	**0.002**
**BMI evolution**					< **0.001**
<27 before diagnosis and <25 five years after diagnosis		70.0	58.7	37.0	
<25 before diagnosis and ≥25 five years after diagnosis		7.2	11.0	21.0	
≥25 before diagnosis and five years after diagnosis		22.8	30.3	42.0	
Healthier diet 5 years after diagnosis		41.0	32.4	29.0	**0.012∗**
PA before diagnosis	Regular	62.9	30.0	14.3	< **0.001**
	Occasional	37.1	65.5	20.7	
	None	0	4.5	65.0	

PA = physical activity.

NCM=Non-Conventional Medicine.

BMI = body mass index (<25 = underweight or normal weight; ≥25 = overweight).

Because of the small numbers, women who were slightly overweight before diagnosis (BMI<27) but had a normal BMI at 5 years were aggregated with those who had a normal weight before diagnosis and 5 years after diagnosis.

PTGI = Post Traumatic Growth Inventory. Higher score indicates higher level of post-traumatic growth.

ERPR+: estrogen receptor positive and/or progesterone receptor positive; Her2: Human epidermal growth factor receptor 2.

∗Mann-Kendall test/∗∗N = 650.

**Table 3:** Factors associated with physical activity five years after breast cancer diagnosis – VICAN 5 survey (n = 706, multinomial logistic regression).

	Regular PA		Occasional PA	
	aOR	95 % CI	aOR	95 % CI
Age at diagnosis	1.00	0.97–1.04	1.01	0.98–1.04
Mental Quality of Life score	**1.04**	**1.01–1.07**	1.01	0.98–1.04
Depressive disorders				
No	**3.45**	**1.22–9.77**	**2.50**	**1.20–5.20**
Yes	1 (ref)		1(ref)	
BMI evolution a				
BMI<25 before diagnosis and ≥25 five years after diagnosis	1(ref)		1(ref)	
BMI<27 before diagnosis and <25 five years after diagnosis	**4.50**	**1.61–12.54**	**2.63**	**1.15–6.05**
BMI ≥25 before diagnosis and five years after diagnosis	1.55	0.51–4.72	1.33	0.55–3.17
PTGI score	**1.03**	**1.01–1.04**	**1.02**	**1.01–1.03**
Arm mobility				
Impaired	1(ref)		1(ref)	
Normal	**3.40**	**1.69–6.85**	1.63	0.87–3.03
NCM use				
No	1 (ref)		1(ref)	
Yes	1.95	0.92–4.12	**1.94**	**1.01–3.78**

PA: physical activity.

PTGI: Post Traumatic Growth Inventory.

NCM: Non-Conventional Medicine.

aOR: adjusted Odds Ratio.

CI: Confidence Interval.

a BMI: Body Mass Index (<25 = underweight or normal weight; ≥25 = overweight). Because of their small numbers, women who were slightly overweight before diagnosis (BMI<27) but had a normal BMI at 5 years were aggregated with those who had a normal BMI before diagnosis and 5 years after diagnosis.

### 3.2 Changes in body weight

The majority of 721 women (missing data n = 2) (62.2 %) had stable BW five years after cancer diagnosis, while 10.4 and 27.4 % had significant weight loss (SWL) and significant weight gain (SWG), respectively. According to BMI criteria, 41.6 % of women were overweight five years after BC diagnosis, including 30.1 % who were also overweight before diagnosis. Over one third of the study sample had adopted a healthier diet, taking into account advice from the media (56.4 %), health professionals (33.2 %) and relatives (26.1 %). Table 4 describes factors associated with SWL and SWG in the univariate analysis. No association was found between BW change and cancer treatment. SWL was positively associated with older age, a lower education level, being retired, a higher BW before diagnosis. It was negatively associated with breast reconstruction. SWG was associated with younger age, lymphedema and impaired arm mobility. In the multinomial logistic regression model (Table 5), SWG since cancer diagnosis was more frequent in younger women, those with a higher BW before diagnosis, and those suffering from lymphedema. Moreover, SWL since cancer diagnosis was only associated with higher BW at diagnosis.

**Table 4:** Factors associated with changes in body weight five years after breast cancer diagnosis – VICAN 5 survey (n = 721, univariate analysis).

		Stable body weight (%) n = 449	Weight loss ≥ 5 kgs (%) n = 75	Weight gain ≥5 kgs (%) n = 197	p-value
Age at diagnosis	*Mean (SD)*	51.0 (11.5)	54.1 (11.2)	47.9 (8.9)	<0.001
Education level	No diploma	4.3	11.1	8.0	0.036
	Primary/Secondary	41.2	48.5	32.5	
	University	54.5	40.4	59.5	
Professional situation	Active	59.5	48.9	59.6	0.021
	Inactive, on sick leave or disability	14.6	12.1	22.2	
	Retired	25.9	39.0	18.2	
Suffered from attitudes of rejection or discrimination		15.1	15.2	22.5	0.141
Perceived financial situation	Comfortable/Getting by Must be careful/Difficult to make ends meet	45.6	40.5	37.2	0.229
		54.4	59.5	62.8	
Breast cancer subtypes b	in situ	12.0	5.1	7.6	0.402
	ERPR+/Her2-	58.8	65.7	67.6	
	Her2+	11.1	8.4	11.9	
	Triple negative	11.7	13.9	10.4	
	Unclassifiable	6.5	6.9	2.4	
Cancer treatment	Chemotherapy	55.0	56.7	58.1	0.823
	Radiotherapy	84.7	86.0	89.9	0.284
	Adjuvant endocrine therapy	65.5	63.3	71.6	0.360
Breast reconstruction		30.3	14.0	30.4	**0.040**
Comorbidity score	*Mean (SD)*	0.65 (0.33)	0.81 (0.38)	0.75 (0.34)	< **0.001**
Medical follow-up for high blood pressure		16.1	29.2	17.1	0.076
Physical Quality of Life	*Mean (SD)*	45.9 (9.7)	43.7 (8.1)	44.4 (9.7)	0.127
Mental Quality of life	*Mean (SD)*	43.7 (10.8)	45.5 (11.8)	41.9 (11.7)	0.054
Uncomfortable with physical appearance		33.5	41.5	43.6	0.089
Lymphedema		31.2	22.2	46.2	**0.001**
Impaired arm mobility		44.1	40.5	59.5	**0.004**
Body weight before diagnosis	*Mean (SD)*	62.2 (11.8)	78.3 (13.3)	64.7 (11.7)	< **0.001**
No physical activity^a^		10.7	15.7	18.7	0.149
Healthier diet^a^		34.6	38.2	30.6	0.547
No alcohol consumption^a^		22.9	24.0	23.0	0.712
Current smoker^a^		22.9	10.1	21.4	0.091

PA = physical activity/NCM = non-conventional medicine/PTGI = Post Traumatic Growth Inventory. Higher score indicates higher level of post-traumatic growth.

ERPR+: estrogen receptor positive and/or progesterone receptor positive; Her2: Human epidermal growth factor receptor 2.

a 5 years after diagnosis.

b N = 657.

**Table 5:** Factors associated with body weight evolution in the five years after breast cancer diagnosis – VICAN 5 survey (n = 721, multinomial logistic regression).

Reference: stable body weight		Weight gain ≥5 kg		Weight loss ≥ 5 kg	
		aOR	95 % CI	aOR	95 % CI
**Age at diagnosis (*per one year increase*)**		**0.97**	**0.95–0.99**	1.01	0.98–1.04
Adjuvant endocrine therapy	Yes	1.23	0.80–1.90	0.95	0.48–1.89
	No	1		1	
**Body weight before diagnosis (*per one kg increase*)**		**1.02**	**1.01–1.04**	**1.08**	**1.05–1.10**
Lymphedema	No	1		1	
	Yes	**1.61**	**1.06–2.44**	0.52	0.25–1.10

aOR: adjusted Odds Ratio.

CI: Confidence Interval.

## 4 Discussion

Decreased PA and weight gain were commonly observed in our representative study sample of French BC survivors five years after diagnosis and were independently associated with impaired mental health and arm lymphoedema, respectively.

### 4.1 Physical activity

A quarter of BC survivors with no cancer progression in the five years after diagnosis reported regular PA. This is far from the expected 100 % given that guidelines recommend systematic PA in patients with cancer. However, it is consistent with the international literature. Previous surveys highlighted that only 31.0–40.0 % of BC survivors interviewed two to ten years after their cancer diagnosis followed PA guidelines [30,31]. In other surveys objectively measuring PA, long-term adherence by women with BC to current recommendations was even lower, from 9.0 to 16.0 % [32,33]. Some studies have highlighted that although adherence to PA recommendations by women with BC was low, it was similar to adherence observed in women with no cancer history [34]. However, in the present study, compared with the pre-diagnosis situation, over half of our study sample had decreased or completely stopped PA five years later. No PA was strongly associated with depressive disorders in our study, while women who reported regular PA had higher mental QoL scores than others. These results reflect those from other surveys which identified mental health difficulties as barriers to adopting and maintaining PA over time in cancer survivors [35,36]. Conversely, a positive psychological change after the traumatic experience of cancer diagnosis seemed to facilitate long-term adherence to PA in our study. The average PTGI in our sample (mean = 57.4) was comparable with that observed in studies on BC survivors in the USA (mean = 64.1) [37] and in France (mean = 59.9) [38]. We found a strong association between PA and higher levels of post-traumatic growth, echoing findings for long-term disease-free Italian cancer survivors, irrespective of cancer site [39]. Although some results in the literature have suggested that PA may lead to better adaptation to cancer experience [40], in our study, we were not able to determine whether PA by itself improved post-traumatic growth, or whether those who experienced higher post-traumatic growth were already more likely to exercise regularly. Indeed, while some items in the PTGI reflect a will to change things in life (e.g., ‘I am more likely to try to change things which need to be changed’), others reflect the consequences of such changes (e.g., ‘I discovered that I am stronger than I thought I was’).

Healthy BW five years after cancer diagnosis and stable or decreased BMI over time were also related to PA. Overweightness and obesity have been associated with infrequent PA in the context of cancer prevention and among cancer survivors [33]. In our study sample of women with BC, regular PA was associated with normal arm mobility, and occasional PA with NCM use. Impaired arm mobility appears to be a major barrier to regular PA in the BC population. This may be linked to fear of worsening pain and lymphedema symptoms, although adapted exercises do exist which can help reduce pain and improve arm mobility [6]. Such exercises need to be implemented early to maximize their benefit [41]. Five years after diagnosis, impaired arm mobility and limitations in performing everyday activities were still present in over half the women who reported occasional PA practice, suggesting insufficient rehabilitation interventions after cancer treatment. For these women, NCM may be an answer to the failure of conventional management of sequelae and serve as a complementary means of coping with impaired health [42]. The positive impact of NCM has previously been described, especially on nausea, pain, fatigue, upper limb functional mobility, anger and anxiety [6]. Moreover, our results are consistent with other studies which highlighted associations between post-traumatic growth following cancer and complementary medicine [43]. Finally, unlike other studies, we did not find any association between PA behavior and breast cancer treatment history [33], the presence of comorbidities [32], or low socioeconomic status [44].

### 4.2 Body weight evolution

In our study sample, 41.6 % of women were overweight five years after cancer diagnosis, while 27.4 % reported at least a 5 kg weight gain compared with their pre-diagnosis BW. Weight gain is a common problem in the literature for many cancer survivors. According to a review conducted by Vance et al. [45], 50–96 % of women experience weight gain during breast cancer treatment, many reporting progressive weight gain in the months and years following diagnosis. Our results highlighted that younger survivors and women with a higher BW before diagnosis were more likely to have gained weight five years later. This echoes findings in other surveys [46,47]. We also found a significant association between weight gain and lymphedema. This too is in line with previous studies; one showed an association between weight gain and pain over long-term survivorship in a large sample of BC survivors [48], and the other an association between weight gain and physical functional limitations two years after BC diagnosis [49]. Weight gain was not associated with PA, although lymphedema and its functional limitations may be considered real barriers to PA. Finally, unlike other studies, we did not find any association between weight gain and chemotherapy [50]. Two possible reasons for this are that our study sample of BC survivors (with no cancer recurrence or progression) was quite homogeneous in terms of care, and because the study took place five years after diagnosis, a relatively long time after the end of chemotherapy. Neither was weight gain associated with adjuvant endocrine therapy, such as in previous studies [50].

Only 10.4 % of our study sample had weight loss of at least 5 kg. Considering that women with progressive diseases were excluded from our analysis, this substantial loss was probably related to voluntary restrictive diets that mostly occurred in women who were overweight at cancer diagnosis. This hypothesis reflects another from a different French survey which suggested that restrictive diets aimed at weight loss are widely practiced by cancer survivors, especially by women with BC [51]. However, unlike other studies, we did not find any association between weight loss and PA [52].

### 4.3 Strengths and limitations

We chose to separately study changes in weight and PA, as it would have been difficult to take into account all the associations between specific health behaviours, weight change and PA in a single analysis. Moreover, we focused on cancer-related associated factors, even though the health component alone is insufficient to obtain an in-depth understanding of the complexity of these two phenomena, as they are also underpinned by social, political, and cultural factors [53,54]. The major strength of our study was its national representativeness. Moreover, our analyses were performed using detailed and reliable data from three sources of data (patient-reported outcomes, medical records and medico-administrative databases). Both VICAN surveys share the general limitations of any approach using self-reported data from questionnaires. Elsewhere, over-reporting was shown in women with BC who self-reported PA frequency [55]. Moreover, eligibility in the survey was restricted to patients able to answer a phone questionnaire in French and we had no information about ethnicity of the participants. In addition, the CATI-based data collection may have introduced social desirability bias resulting in an underestimation of respondents’ weight gain and an overestimation of their PA frequency.

## 5 Conclusion

PA improves overall prognosis in long-term BC survivors, even in those insufficiently active pre-diagnosis [3]. Survivorship guidelines all insist on the importance of encouraging PA in all women after BC diagnosis. PA should be personalized and adapted to co-morbidities, clinical status, physical abilities, and patient preferences. Our results highlight the strong association between long-term (5 years) adherence to PA and both mental health well-being and the lack of impairment in arm mobility in French BC survivors. The systematic detection and management of depressive disorders, education about the importance of healthy BW, and adapted sequelae management, are all essential from the beginning of patient care and should be continued during long-term follow-up to help BC survivors engage in and maintain a healthy lifestyle.

## Ethical approval

The methodology was approved by the following three national ethics commissions: the CCTIRS (Advisory Committee on the Processing of Information in the Field of Health Research, registration number 11–143), the ISP (Institute of Public Health, registration number C11-63), and the CNIL (French Commission on Individual Data Protection and Public Liberties, registration number 911290).

## Declaration of competing interest

None.
